# Mycorrhizal Symbionts and Associated Bacteria: Potent Allies to Improve Plant Phosphorus Availability and Food Security

**DOI:** 10.3389/fmicb.2021.797381

**Published:** 2022-01-10

**Authors:** Cristiana Sbrana, Monica Agnolucci, Luciano Avio, Luca Giovannini, Michela Palla, Alessandra Turrini, Manuela Giovannetti

**Affiliations:** ^1^National Research Council (CNR), Institute of Agricultural Biology and Biotechnology, Pisa, Italy; ^2^Department of Agriculture, Food and Environment, University of Pisa, Pisa, Italy

**Keywords:** beneficial soil microbiota, arbuscular mycorrhizal symbionts, phosphate solubilizing bacteria, phytate mineralizing bacteria, phosphate transporter genes

## Introduction

Phosphorus (P) is an essential nutrient for food production and a main component of nitrogen-phosphorus-potassium fertilizers used for the cultivation of food crops worldwide. The most significant source of P is phosphate rock, which is mined globally (223 million tons in 2020) in only a few countries, including China (90), Morocco and Western Sahara (37), USA (24) and Russia (13) (U.S. Geological Survey, 2021). As a consequence, the European Union and most world countries are almost entirely dependent on imports of phosphate rock, making them vulnerable to disruptions in its supply and undermining their food security. Moreover, phosphate rock is a finite, non-renewable resource, whose reserves are estimated to be depleted within 100–200 years (Cordell and White, 2014).

In high-intensity agriculture, starting in the mid-20th century with the green revolution, the increased use of P fertilizers has been linked to diverse environmental effects, encompassing eutrophication, the loss of landscape quality, greenhouse gas emissions, excessive fresh-water consumption and cadmium accumulation in food plants (Chen and Graedel, 2016). Yet, there are no substitutes for P in food production systems, as P is essential to life. Indeed, P is a structural component of DNA and RNA (in the form of orthophosphate ions ${\text{PO}}_{4}^{3-}$), plays a key role in energy transfer as an element of ATP, and is a building component of biomolecules involved in important biological processes, such as photosynthesis, phospholipid biosynthesis and respiration. The extensive use of P fertilizers has led to very high concentrations of P in soils, but the soluble fraction (${\text{PO}}_{4}^{3-}$), which is the only form directly available for plant nutrition, represents a very small fraction because more than 80% of soil P is insoluble and therefore unavailable for plant uptake. Such dominant P form is represented by organic and mineral P, due to its immobilization in organic matter and precipitation with other soil minerals, i.e., iron (Fe) and aluminum (Al) in acid soils and calcium (Ca) in alkaline soils. Thus, the issue of improving a more efficient use of P is becoming increasingly topical, and diverse potential measures have been considered, in order to reduce P fertilizers in agriculture, while maintaining crop yields and minimizing their environmental impact. Such measures range from optimizing land use by crop rotations, preventing erosion, maintaining soil quality and organic matter, improving fertilizer recommendations and crop genotypes, extending plant root P uptake capacity by means of beneficial microorganisms, primarily mycorrhizal symbionts (Schröder et al., 2011). This opinion article focuses on the current knowledge and prospects for the role of mycorrhizal fungi and their associated bacteria in P availability to food plants.

## Arbuscular Mycorrhizal Symbioses and Soil P Uptake

**Figure 1:**
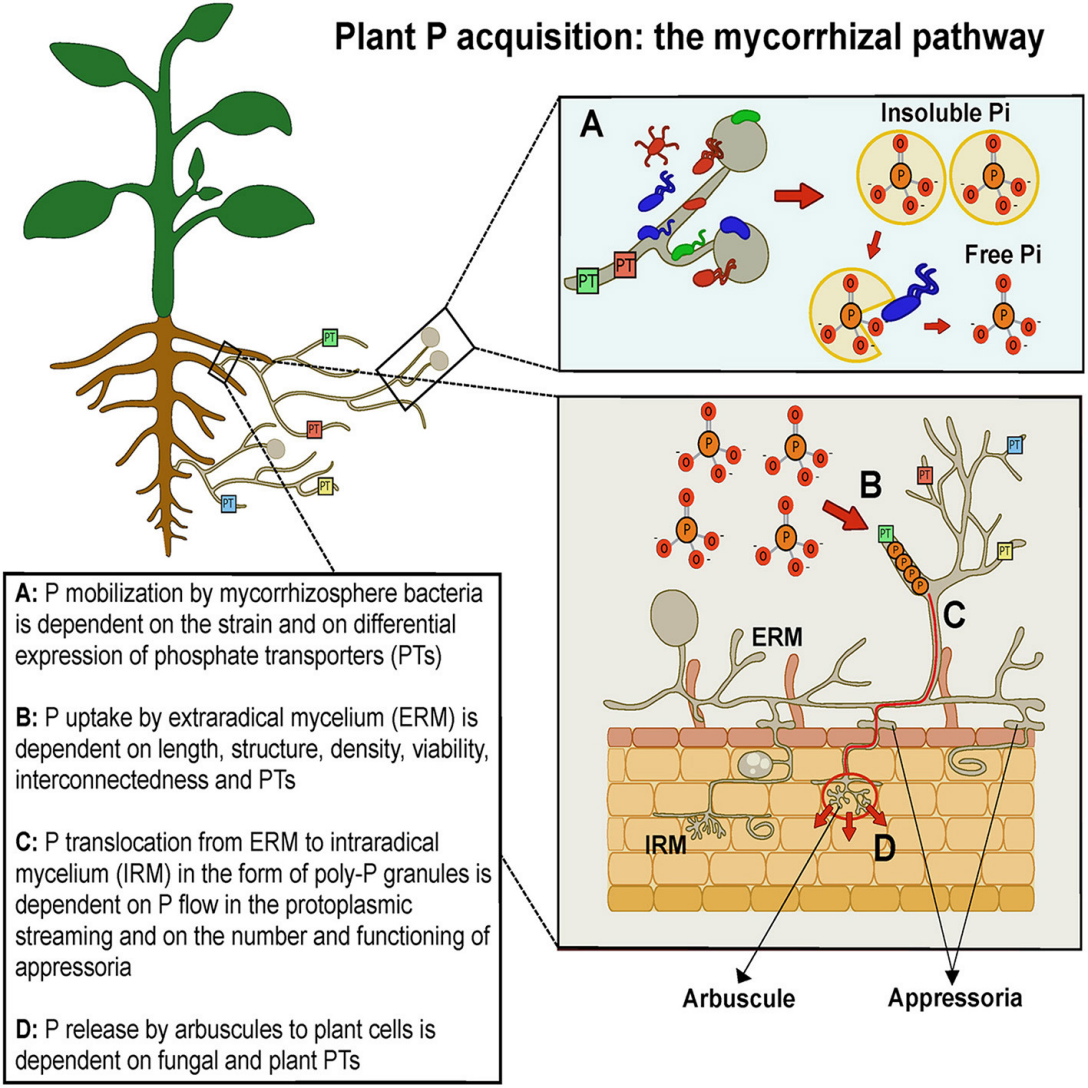
Schematic drawing representing phosphate pathway from soil to plant via arbuscular mycorrhizal fungi and mycorrhizosphere bacteria.

Arbuscular mycorrhizal (AM) fungi (AMF), belonging to Mucoromycota, sub-phylum Glomeromycotina (Spatafora et al., 2016), establish beneficial symbiotic associations with the roots of most land plants, including major staple food crops. AMF improve plant nutrition and tolerance to biotic and abiotic stresses and are key organisms in soil nutrient cycles and in the maintenance of biological soil fertility (Smith and Read, 2008). Moreover, AMF affect plant secondary metabolism, enhancing the biosynthesis of health-promoting phytochemicals, such as polyphenols and carotenoids (Agnolucci et al., 2020). In this mutualistic symbiosis AMF obtain photosynthesis-derived carbon (up to 20%) in exchange for soil mineral nutrients, whose uptake and translocation is facilitated by a fine network of extraradical hyphae functioning as an auxiliary absorbing system (Figure 1). The direct uptake of P by roots is rapid and results in P depletion zones because P mobility in soil is low; however, the extraradical mycelium (ERM) of AMF spread well beyond root P depletion zones (Smith and Read, 2008). Thus, ERM represents an important variable affecting fungal absorbing surface and foraging ability, and therefore the rate of plant P uptake from the soil. Using *in vitro* monoxenic cultures, ERM was unequivocally demonstrated to be able to hydrolyse organic phosphate and release acid phosphatase, facilitating P mobilization (Koide and Kabir, 2000; Sato et al., 2019). In different AMF isolates, ERM density ranges between 2.7 and 20.5 m g^−1^ of soil, with a mean growth rate of 0.74–1.1 m d^−1^ (Giovannetti et al., 2001; Mikkelsen et al., 2008). Actually, ERM length was found to be positively correlated with plant shoot biomass and P content in different plants, including clover and maize (Jakobsen et al., 1992; Sawers et al., 2017), while the production of large numbers of appressoria—the structures connecting ERM to intraradical mycelium (IRM)—showed significant positive correlations with shoot and plant P content (Pepe et al., 2020). However, great intraspecific differences were detected among AMF species and isolates in ERM extent, biomass and viability, affecting plant growth responses (Munkvold et al., 2004; Pepe et al., 2017, 2018).

## Fungal P Transporters, P Translocation and Transfer to Plant Cells

Beyond ERM structural and functional traits, P uptake is facilitated by fungal P transporters (PTs), whose occurrence and differential expression affect plant P absorption from the soil solution and nutrition. P is absorbed from the soil in the form of ${\text{PO}}_{4}^{3-}$, a negatively charged ion requiring metabolic energy and high-affinity PTs for its transport across the fungal plasma membrane (Ferrol et al., 2019). A pioneering study detected the first gene encoding a trans-membrane phosphate transporter (GvPT), which was predominantly expressed in ERM and supposed to play a role in P uptake from the soil by the AM symbiont *Glomus versiforme* (Harrison and van Buuren, 1995). Other PT genes, *GintPT* and *GmosPT*, expressed in *Glomus intraradices* and *Glomus mosseae* extraradical and intraradical hyphae, respectively, were found to be regulated in the response to P concentrations in ERM environment (Maldonado-Mendoza et al., 2001; Benedetto et al., 2005; Fiorilli et al., 2013). Recent studies highlighted that four different *Rhizophagus irregularis* PTs, expressed both in ERM and IRM, were modulated by P availability in microcosm systems (Walder et al., 2016; Calabrese et al., 2019). Interestingly, a PT identified in *Gigaspora margarita* was found to be involved also in the P signaling pathway, functioning as a P transceptor (Xie et al., 2016).

P uptake by ERM is followed by P storage in vacuoles as polyphosphate (poly-P) granules, which are translocated through protoplasmic streaming and/or a tubular vacuole system from soil- to root-based hyphae, before being absorbed by plant root cells (Ezawa and Saito, 2018). P flow in ERM is very rapid, given the high rates of hyphal protoplasmic streaming, 3–9 μm s^−1^ (Logi et al., 1998; Giovannetti et al., 2000) and generates P fluxes from extraradical to intraradical hyphae ranging from 1.7 to 4.2 × 10^−8^ mol cm^−2^ s^−1^, depending on AMF species and isolates (Pepe et al., 2020). In intraradical hyphae poly-P is supposed to be hydrolysed by fungal polyphosphatases and then transferred to the plant cell, although the biochemical and molecular mechanisms of such processes are largely unknown (Ferrol et al., 2019).

The transfer of P released by fungal symbionts to plant cells is mediated by specific plant PTs and membrane-integral proton ATPases. The first plant PTs were detected in mycorrhizal potato, rice and *Medicago truncatula*, for example *StPT3* was expressed in potato root cells colonized by *G. intraradices* (Rausch et al., 2001), while *MtPT4* mediated the uptake of P released by the AM symbionts *G. versiforme* and *Gigaspora gigantea* (Harrison et al., 2002). The latter PT was shown to be essential for the acquisition of P released by the fungus and the establishment of the symbiosis (Javot et al., 2007). Indeed, in *Astragalus sinicus*, P transporters *AsPT1* and *AsPT4* were required for the formation of AM symbiosis, as their suppression reduced root colonization and arbuscule development (Xie et al., 2013), while in tomato specific transcripts occurred only in arbuscule-containing cells (Gomez-Ariza et al., 2009). Interestingly, a high plant P status was found to represent a major regulator of PHT1 genes expression (Nagy et al., 2009). Other mycorrhiza-specific PTs were characterized in different plant species, such as rice, petunia, sorghum and flax, as well as H^+^-ATPase genes, whose expression leads to the generation of the proton gradient needed for P uptake (Ferrol et al., 2019). It is important to note that in maize plants colonized by *R. irregularis*, transcripts encoded by *ZmPHT1* genes were correlated with ERM length and P uptake (Sawers et al., 2017), two variables found to be related in previous studies (Jakobsen et al., 1992).

## AMF-Associated Bacteria and Possible Role in P Uptake

Plant P nutrition is boosted also by another component of AMF symbioses, represented by the bacterial communities living closely associated with spores, sporocarps and extraradical mycelium, in the region defined mycorrhizosphere (Barea et al., 2002). The composition of AMF-associated microbiota may depend on both fungal taxon and host plant species (Roesti et al., 2005; Long et al., 2008; Agnolucci et al., 2015). Such bacteria, when isolated in pure culture, showed not only plant growth promoting (PGP) functions and mycorrhiza helper (MH) activities, but also the ability to solubilize P from mineral phosphates and mineralize P from phytates (Giovannini et al., 2020a). Indeed, 26 *Burkholderia* spp. strains were able to solubilize phosphate and to become strongly attached to *R. irregularis* mycelium (Taktek et al., 2015), while 12 out of 128 bacterial strains isolated from *Rhizoglomus irregulare* mycelium showed phosphate-solubilizing activity (Sharma et al., 2020) and 70% of bacteria isolated from *R. irregularis* spores were able to mineralize P (Battini et al., 2016). The mechanism underlying such activities was ascribed to the production of organic acids by AMF and associated microbiota (Andrino et al., 2021). A recent study reported that phosphate solubilising bacteria migrated along extraradical AMF mycelium toward a phytate source, which they were able to mineralise (Jiang et al., 2021). Thus, P-mobilizing bacteria can increase P availability for AMF, therefore playing a key role in AMF P acquisition from soil and plant P nutrition facilitation (Figure 1). Actually, a pioneering work posed the question as to whether selected bacterial strains isolated from AMF spores and showing *in vitro* P-mobilizing activities might be able to show the same specific traits also *in planta*, affecting P nutrition in mycorrhizal and control maize plants. The use of radioactive P allowed the detection of *Streptomyces* sp. W94 and *Streptomyces* sp. W77 as the strains producing the largest increases in the uptake and translocation of ^33^P and the highest enhancement of hyphal length-specific ^33^P uptake, respectively (Battini et al., 2017).

## Concluding Remarks and Future Prospects

The fundamental role of AM symbionts in plant P nutrition has been largely investigated and documented. Though, important questions remain to be answered in order to manipulate the symbiosis and implement AMF inocula in sustainable agroecosystems. First of all, extensive genetic works should be performed on a large number of AMF, given their high genetic and functional diversity, not only at the interspecific but also at the intraspecific level (Koch et al., 2004; Avio et al., 2006; Wyss et al., 2016). Indeed, studies performed on the model species *Rhizophagus irregularis* showed that diverse AMF genotypes differentially interacted with edaphic traits and affected host plant responses (Koch et al., 2006; Angelard et al., 2010; Venegas et al., 2021). It is not yet known whether such differences in plant growth and P uptake are related to the differential ability to absorb P from the soil and translocate it to the host plant. Accordingly, the aim of future works should be to understand the genetic basis of the relationship between ERM structural and functional traits, investigating in particular the differential abundance and expression of P transporter genes in genetically different AMF isolates (Giovannini et al., 2020a).

Systematic studies on the chemical and biochemical events underlying P mobilization by different AMF isolates and mycorrhizosphere bacteria could detect the most efficient combinations showing synergistic activities related to organic acid secretion and phosphatase/phytase enzyme production. Though, another key question remains, as to whether P mobilization from insoluble to soluble forms operated by mycorrhizosphere bacteria can enhance the expression of P transporter genes in ERM hyphae, P uptake and plant P nutrition. In order to utilize the most efficient AMF and associated bacteria consortia as inocula in agriculture, parallel studies are needed on their ability to survive in the new soil environment, to colonize plant roots and to compete with native bacteria and AMF for P scavenging and uptake (Rodriguez and Sanders, 2015; Giovannini et al., 2020b). Such studies could lead to the selection of innovative biofertilizers to be used as inoculants for increasing P acquisition by crop plants, in new and more sustainable food production systems, in the years to come.

## Author Contributions

CS, MA, AT, and MG conceived the topic of the paper and wrote the original draft. LG, MP, and LA participated in the preparation and review of the manuscript. LG and MP provided editing assistance. All authors have read and agreed to the published version of the manuscript.

## Funding

This work was funded by University of Pisa, Italy, Grant: Fondi di Ateneo.

## Conflict of Interest

The authors declare that the research was conducted in the absence of any commercial or financial relationships that could be construed as a potential conflict of interest.

## Publisher's Note

All claims expressed in this article are solely those of the authors and do not necessarily represent those of their affiliated organizations, or those of the publisher, the editors and the reviewers. Any product that may be evaluated in this article, or claim that may be made by its manufacturer, is not guaranteed or endorsed by the publisher.
